# A new polymorph of aqua­bis­(1,10-phenanthroline-κ^2^ *N*,*N*′)copper(II) dinitrate

**DOI:** 10.1107/S1600536814008198

**Published:** 2014-04-18

**Authors:** Mehdi Boutebdja, Asma Lehleh, Adel Beghidja, Zouaoui Setifi, Hocine Merazig

**Affiliations:** a Unité de Recherche de Chimie de l’Environnement et Moléculaire Structurale, (CHEMS), Faculté des Sciences Exactes, Département de Chimie, Université de Constantine 1, 25000 Constantine, Algeria

## Abstract

The title mol­ecule, [Cu(C_12_H_8_N_2_)_2_(H_2_O)](NO_3_)_2_, is a new polymorph of a compound which up to now has been reported to crystallize space groups in *C*2/*c* and *Cc*. The crystal studied was twinned by non-merohedry (final BASF factor of 0.40043) with the structure being solved and refined in *P*-1. The Cu^II^ atom is coordinated by four N atoms from two 1,10-phenanthroline ligands and an O atom from a water mol­ecule in an approximate trigonal–bipyramidal geometry. Discrete entities of one cation and two nitrate anions are formed by water–nitrate O—H⋯O hydrogen bonds. The components are further assembled into a three-dimensional network by C—H⋯O hydrogen bonds.

## Related literature   

For structural analyses of the other polymorphs, see: Nakai & Deguchi (1975 ▶); Catalan *et al.* (1995 ▶); Szpakolski *et al.* (2010 ▶); Zhou (2011 ▶).
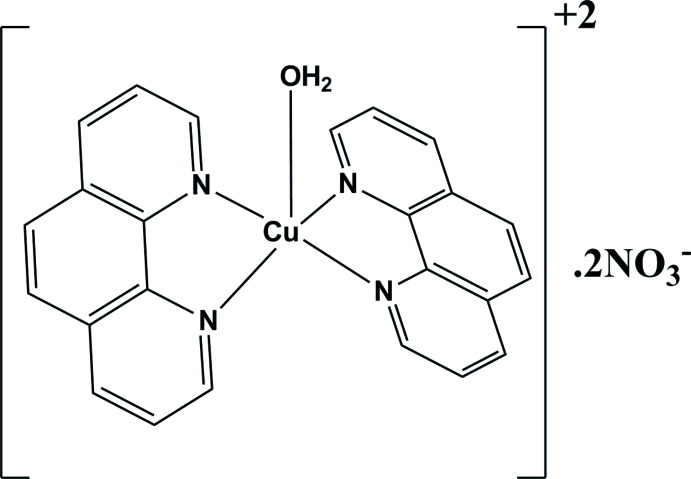


## Experimental   

### 

#### Crystal data  

[Cu(C_12_H_8_N_2_)_2_(H_2_O)](NO_3_)_2_
*M* *_r_* = 565.99
Triclinic, 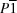

*a* = 7.0836 (3) Å
*b* = 11.7898 (3) Å
*c* = 14.2951 (4) Å
α = 78.079 (2)°
β = 79.862 (3)°
γ = 73.782 (3)°
*V* = 1112.68 (7) Å^3^
*Z* = 2
Mo *K*α radiation
μ = 1.05 mm^−1^
*T* = 150 K
0.12 × 0.10 × 0.08 mm

#### Data collection  

Bruker APEXII CCD diffractometer
17314 measured reflections
11903 independent reflections
10562 reflections with *I* > 2σ(*I*)
*R*_int_ = 0.041

#### Refinement  

*R*[*F* ^2^ > 2σ(*F* ^2^)] = 0.058
*wR*(*F* ^2^) = 0.172
*S* = 1.17
11903 reflections
344 parameters
3 restraints
H-atom parameters constrained
Δρ_max_ = 0.98 e Å^−3^
Δρ_min_ = −0.63 e Å^−3^

### 

Data collection: *APEX2* (Bruker, 2006 ▶); cell refinement: *SAINT* (Bruker, 2006 ▶); data reduction: *SAINT*; program(s) used to solve structure: *SHELXS97* (Sheldrick, 2008 ▶); program(s) used to refine structure: *SHELXL97* (Sheldrick, 2008 ▶); molecular graphics: *ATOMS* (Dowty, 1995 ▶); software used to prepare material for publication: *WinGX* (Farrugia, 2012 ▶).

## Supplementary Material

Crystal structure: contains datablock(s) global, I. DOI: 10.1107/S1600536814008198/im2451sup1.cif

Structure factors: contains datablock(s) I. DOI: 10.1107/S1600536814008198/im2451Isup2.hkl

CCDC reference: 996861

Additional supporting information:  crystallographic information; 3D view; checkCIF report

## Figures and Tables

**Table 1:** Hydrogen-bond geometry (Å, °)

*D*—H⋯*A*	*D*—H	H⋯*A*	*D*⋯*A*	*D*—H⋯*A*
O1*W*—H1*W*⋯O2^i^	0.79	1.92	2.709 (5)	176
O1*W*—H2*W*⋯O6	0.87	1.89	2.718 (5)	159
C2—H2⋯O1^ii^	0.93	2.57	3.313 (5)	137
C5—H5⋯O5^iii^	0.93	2.40	3.271 (5)	156
C6—H6⋯O6^iv^	0.93	2.56	3.417 (6)	154
C8—H8⋯O3^v^	0.93	2.50	3.194 (5)	131
C17—H17⋯O2^vi^	0.93	2.49	3.363 (5)	157
C18—H18⋯O1^vii^	0.93	2.50	3.406 (5)	166
C20—H20⋯O3^vii^	0.93	2.49	3.357 (5)	155

Symmetry codes: (i) 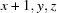; (ii) 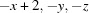; (iii) 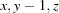; (iv) 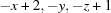; (v) 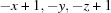; (vi) 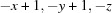; (vii) 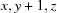.

**Fig. 1.**
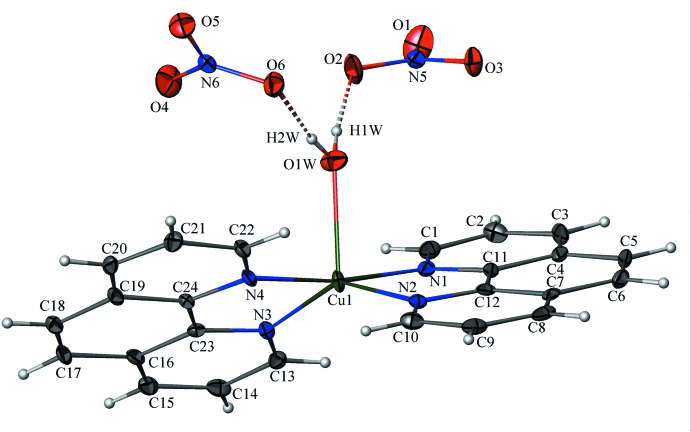
View of the title compound with the atom numbering scheme. Displacement ellipsoids for non-H atoms are drawn at the 30% probability level.

**Fig. 2.**
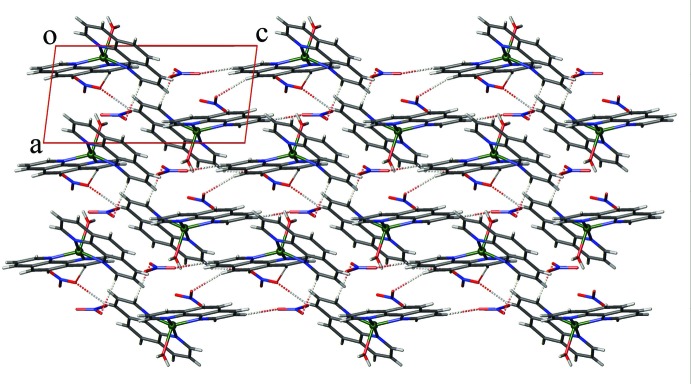
Partial view of the crystal structure of the title compound showing the hydrogen bonds.

## supplementary crystallographic information

### 1. Comment

The reported structure of complex (I) is a polymorph of previously reported material. It crystallizes as a non-merohedral twin in the triclinic system with the space group P1, contrary to what has been observed in other structural analyses which three times report the crystal symmetry to correspond to the space group *C*2/*c* (Nakai & Deguchi (1975); Szpakolski *et al.* (2010); Zhou (2011)), while the fourth crystal structure was reported in the space group *Cc* (Catalan *et al.*, 1995).

Compound (I) has a discrete structure containing monomeric [Cu(H_2_O)(1,10'-phen)]^2+^ cations and two counter-balanced nitrate anions which are connected to the cation *via* O–H···O hydrogen bonds. The Cu(II) ion is coordinated by two 1,10'-phenantroline molecules each acting as a bidentate ligand (through the four nitrogen atoms (N1,N2, N4,N3)) and one water molecule O1w (Fig. 1). The geometry around the metal is of distorted trigonal bipyramidal geometry and all distances are in a normal range. The dihedral angle between the two 1,10'-phenantroline molecules is 34.92 (3)°, while the dihedral angle varies in its analogous between 37.89 (3)° and 53.46 (3)°. In the crystal, molecules are linked by extensive hydrogen bonds involving the nitrate anions and phenantroline and water molecules, producing a three-dimensional network (Fig 2).

### 2. Experimental

A methanolic solution containing Cu(NO_3_)_2_ × 3 H_2_O (0.1208 g, 0.5 mmol) was added with stirring to a methanolic solution containing 1,10'-phenantroline (0.9 g, 0.5 mmol). After a few minutes a blue green precipitate appears and was filtrated. The blue green filtrate was kept for several weeks at room temperature. Green crystals suitable for X-ray analysis were obtained (yield: 0.20 g, 70% on the basis of Cu(NO_3_)_2_.3H_2_O).

### 3. Refinement

Water hydrogen atoms were tentatively found in the difference density Fourier map and were refined with an isotropic displacement parameter of *U*_iso_(H) = 1.5 *U*_eq_(O1W). O—H distances were restrained to be 0.9 Å within a standard deviation of 0.01 and the H···H contacts were restraint to 1.40 Å with a standard deviation of 0.02. A l l other hydrogen atoms were placed in calculated positions with C —H distances of 0.93–0.96 Å for aromatic H atoms with *U*_iso_(H) =1.2 *U*_eq_(C).

The presence of a non-merohedral twin was identified using TwinRotMat within PLATON (Spek, 2009) (twin law: (0.958 0.013 0.071), (0.979 -0.994 0.036), (0.979 0.006 -0.964)) reducing the conventional R-factor from 0.11 to 0.057, with a final BASF factor (HKLF 5 format) of 0.40043. Maximum and minimum residual electron densities were 0.98 eÅ-3 (0.95 Å from Cu01) and -0.63 eÅ-3 (0.93 Å from Cu01 ), respectively.

### Crystal data

**Table:**

[Cu(C_12_H_8_N_2_)_2_(H_2_O)](NO_3_)_2_	*Z* = 2
*M**_r_* = 565.99	*F*(000) = 578
Triclinic, *P*1	*D*_x_ = 1.689 Mg m^−^^3^
Hall symbol: -P 1	Mo *K*α radiation, λ = 0.71073 Å
*a* = 7.0836 (3) Å	Cell parameters from 11062 reflections
*b* = 11.7898 (3) Å	θ = 1.8–34.6°
*c* = 14.2951 (4) Å	µ = 1.05 mm^−^^1^
α = 78.079 (2)°	*T* = 150 K
β = 79.862 (3)°	Block, green
γ = 73.782 (3)°	0.12 × 0.10 × 0.08 mm
*V* = 1112.68 (7) Å^3^	

### Data collection

**Table:**

Bruker APEXII CCD diffractometer	10562 reflections with *I* > 2σ(*I*)
Radiation source: Rotating Anode	*R*_int_ = 0.041
Graphite monochromator	θ_max_ = 27.9°, θ_min_ = 2.1°
Detector resolution: 18.4 pixels mm^-1^	*h* = −9→9
φ and ω scans	*k* = −15→15
17314 measured reflections	*l* = −18→18
11903 independent reflections	

### Refinement

**Table:**

Refinement on *F*^2^	3 restraints
Least-squares matrix: full	Hydrogen site location: mixed
*R*[*F*^2^ > 2σ(*F*^2^)] = 0.058	H-atom parameters constrained
*wR*(*F*^2^) = 0.172	*w* = 1/[Σ^2^(*F*O^2^) + (0.0916*P*)^2^ + 1.1415*P*] where *P* = (*F*O^2^ + 2*F*C^2^)/3
*S* = 1.17	(Δ/σ)_max_ = 0.001
11903 reflections	Δρ_max_ = 0.98 e Å^−^^3^
344 parameters	Δρ_min_ = −0.63 e Å^−^^3^

### Special details

**Table:**

Geometry. Bond distances, angles etc. have been calculated using the rounded fractional coordinates. All su's are estimated from the variances of the (full) variance-covariance matrix. The cell esds are taken into account in the estimation of distances, angles and torsion angles
Refinement. Refinement on F^2^ for ALL reflections except those flagged by the user for potential systematic errors. Weighted R-factors wR and all goodnesses of fit S are based on F^2^, conventional R-factors R are based on F, with F set to zero for negative F^2^. The observed criterion of F^2^ > 2sigma(F^2^) is used only for calculating -R-factor-obs etc. and is not relevant to the choice of reflections for refinement. R-factors based on F^2^ are statistically about twice as large as those based on F, and R-factors based on ALL data will be even larger.

### Fractional atomic coordinates and isotropic or equivalent isotropic displacement parameters (Å^2^)

**Table:**

	*x*	*y*	*z*	*U*_iso_*/*U*_eq_	
Cu01	0.88124 (7)	0.26741 (4)	0.24554 (3)	0.0110 (1)	
O1W	1.1790 (4)	0.2580 (3)	0.2736 (2)	0.0211 (9)	
N1	0.9959 (5)	0.0992 (3)	0.2189 (2)	0.0107 (8)	
N2	0.7695 (5)	0.1813 (3)	0.3734 (2)	0.0102 (8)	
N3	0.8143 (5)	0.3546 (3)	0.1125 (2)	0.0108 (8)	
N4	0.8066 (5)	0.4349 (3)	0.2743 (2)	0.0106 (8)	
C1	1.1142 (6)	0.0599 (3)	0.1420 (3)	0.0138 (10)	
C2	1.2069 (6)	−0.0618 (4)	0.1400 (3)	0.0166 (11)	
C3	1.1773 (6)	−0.1452 (3)	0.2200 (3)	0.0157 (11)	
C4	1.0526 (6)	−0.1077 (3)	0.3020 (3)	0.0123 (10)	
C5	1.0093 (6)	−0.1886 (3)	0.3887 (3)	0.0158 (11)	
C6	0.8865 (6)	−0.1471 (4)	0.4654 (3)	0.0165 (11)	
C7	0.8004 (6)	−0.0215 (3)	0.4637 (3)	0.0130 (10)	
C8	0.6764 (6)	0.0268 (4)	0.5424 (3)	0.0165 (11)	
C9	0.6022 (6)	0.1487 (4)	0.5337 (3)	0.0159 (11)	
C10	0.6507 (6)	0.2242 (4)	0.4478 (3)	0.0144 (10)	
C11	0.9666 (5)	0.0160 (3)	0.2980 (3)	0.0106 (10)	
C12	0.8408 (5)	0.0607 (3)	0.3808 (3)	0.0102 (9)	
C13	0.8086 (6)	0.3120 (3)	0.0336 (3)	0.0129 (10)	
C14	0.7705 (6)	0.3877 (4)	−0.0548 (3)	0.0147 (11)	
C15	0.7352 (6)	0.5097 (4)	−0.0617 (3)	0.0143 (10)	
C16	0.7332 (5)	0.5576 (3)	0.0216 (3)	0.0115 (10)	
C17	0.6900 (6)	0.6839 (3)	0.0236 (3)	0.0146 (10)	
C18	0.6955 (6)	0.7250 (3)	0.1053 (3)	0.0143 (10)	
C19	0.7375 (5)	0.6431 (3)	0.1932 (3)	0.0120 (10)	
C20	0.7436 (6)	0.6794 (3)	0.2806 (3)	0.0133 (10)	
C21	0.7790 (6)	0.5936 (3)	0.3618 (3)	0.0145 (11)	
C22	0.8106 (6)	0.4717 (3)	0.3562 (3)	0.0125 (10)	
C23	0.7737 (5)	0.4760 (3)	0.1066 (3)	0.0099 (9)	
C24	0.7716 (5)	0.5196 (3)	0.1939 (3)	0.0103 (10)	
O1	0.6976 (6)	0.0152 (3)	0.0982 (2)	0.0341 (11)	
O2	0.5277 (5)	0.1630 (3)	0.1717 (2)	0.0251 (9)	
O3	0.5629 (5)	−0.0219 (3)	0.2460 (2)	0.0229 (9)	
N5	0.5964 (5)	0.0505 (3)	0.1718 (2)	0.0143 (9)	
O4	1.2876 (6)	0.5301 (3)	0.2707 (2)	0.0291 (10)	
O5	1.3245 (5)	0.5481 (3)	0.4138 (2)	0.0242 (9)	
O6	1.2436 (5)	0.3916 (3)	0.3929 (2)	0.0204 (8)	
N6	1.2854 (5)	0.4914 (3)	0.3584 (2)	0.0143 (9)	
H1	1.13600	0.11560	0.08750	0.0170*	
H1W	1.28200	0.23300	0.24360	0.0220*	
H2	1.28740	−0.08550	0.08510	0.0200*	
H2W	1.19590	0.31520	0.29900	0.0220*	
H3	1.23910	−0.22600	0.22000	0.0190*	
H5	1.06640	−0.27040	0.39210	0.0190*	
H6	0.85780	−0.20120	0.51990	0.0200*	
H8	0.64570	−0.02320	0.59910	0.0200*	
H9	0.51940	0.18170	0.58470	0.0190*	
H10	0.59810	0.30650	0.44320	0.0170*	
H13	0.83060	0.22980	0.03720	0.0150*	
H14	0.76920	0.35510	−0.10850	0.0180*	
H15	0.71300	0.56000	−0.12020	0.0170*	
H17	0.65770	0.73840	−0.03180	0.0180*	
H18	0.67180	0.80690	0.10420	0.0170*	
H20	0.72390	0.76000	0.28340	0.0160*	
H21	0.78200	0.61630	0.42000	0.0170*	
H22	0.83510	0.41490	0.41130	0.0150*	

### Atomic displacement parameters (Å^2^)

**Table:**

	*U*^11^	*U*^22^	*U*^33^	*U*^12^	*U*^13^	*U*^23^
Cu01	0.0172 (3)	0.0060 (2)	0.0083 (2)	−0.0009 (2)	−0.0024 (2)	0.0000 (2)
O1W	0.0146 (14)	0.0201 (15)	0.0317 (16)	−0.0024 (12)	−0.0021 (12)	−0.0148 (13)
N1	0.0125 (15)	0.0102 (15)	0.0102 (14)	−0.0040 (12)	−0.0032 (11)	−0.0002 (11)
N2	0.0110 (15)	0.0111 (15)	0.0096 (14)	−0.0031 (12)	−0.0049 (11)	−0.0010 (11)
N3	0.0130 (15)	0.0092 (14)	0.0099 (14)	−0.0026 (12)	−0.0022 (11)	−0.0005 (11)
N4	0.0115 (15)	0.0081 (14)	0.0108 (14)	−0.0005 (12)	−0.0027 (11)	0.0002 (11)
C1	0.0154 (18)	0.0138 (18)	0.0122 (17)	−0.0034 (15)	−0.0023 (14)	−0.0022 (14)
C2	0.0155 (19)	0.0184 (19)	0.0164 (19)	−0.0011 (15)	−0.0027 (15)	−0.0077 (15)
C3	0.0165 (19)	0.0092 (17)	0.022 (2)	0.0004 (15)	−0.0066 (15)	−0.0054 (14)
C4	0.0140 (18)	0.0093 (17)	0.0161 (18)	−0.0052 (14)	−0.0070 (14)	0.0000 (13)
C5	0.0183 (19)	0.0083 (17)	0.023 (2)	−0.0062 (15)	−0.0109 (16)	0.0033 (14)
C6	0.020 (2)	0.0142 (18)	0.0170 (19)	−0.0097 (16)	−0.0078 (15)	0.0058 (14)
C7	0.0124 (17)	0.0143 (18)	0.0148 (18)	−0.0077 (14)	−0.0068 (14)	0.0025 (14)
C8	0.0141 (18)	0.027 (2)	0.0106 (17)	−0.0117 (17)	−0.0031 (14)	0.0020 (15)
C9	0.0128 (18)	0.025 (2)	0.0106 (17)	−0.0062 (16)	−0.0013 (14)	−0.0027 (15)
C10	0.0130 (17)	0.0174 (19)	0.0133 (17)	−0.0031 (15)	−0.0032 (14)	−0.0034 (14)
C11	0.0109 (17)	0.0102 (17)	0.0113 (16)	−0.0029 (14)	−0.0047 (13)	−0.0003 (13)
C12	0.0094 (16)	0.0114 (17)	0.0108 (16)	−0.0042 (14)	−0.0038 (13)	0.0003 (13)
C13	0.0138 (18)	0.0114 (17)	0.0137 (18)	−0.0034 (14)	−0.0022 (14)	−0.0022 (14)
C14	0.0154 (18)	0.020 (2)	0.0095 (17)	−0.0039 (16)	−0.0043 (14)	−0.0026 (14)
C15	0.0150 (18)	0.0174 (19)	0.0100 (17)	−0.0040 (15)	−0.0044 (14)	0.0014 (14)
C16	0.0084 (16)	0.0144 (18)	0.0107 (17)	−0.0031 (14)	−0.0033 (13)	0.0023 (13)
C17	0.0138 (18)	0.0118 (18)	0.0149 (18)	−0.0025 (15)	−0.0031 (14)	0.0054 (14)
C18	0.0116 (17)	0.0097 (17)	0.0188 (19)	−0.0003 (14)	−0.0023 (14)	0.0007 (14)
C19	0.0092 (16)	0.0096 (17)	0.0148 (17)	−0.0001 (14)	−0.0005 (13)	−0.0010 (13)
C20	0.0129 (17)	0.0083 (17)	0.0188 (19)	−0.0026 (14)	−0.0006 (14)	−0.0039 (14)
C21	0.0178 (19)	0.0132 (18)	0.0137 (18)	−0.0031 (15)	−0.0019 (14)	−0.0058 (14)
C22	0.0146 (18)	0.0098 (17)	0.0118 (17)	−0.0024 (14)	−0.0029 (14)	0.0009 (13)
C23	0.0080 (16)	0.0094 (16)	0.0116 (17)	−0.0018 (13)	−0.0019 (13)	−0.0004 (13)
C24	0.0086 (16)	0.0098 (17)	0.0117 (17)	−0.0008 (14)	−0.0033 (13)	−0.0001 (13)
O1	0.050 (2)	0.0320 (19)	0.0223 (16)	−0.0173 (17)	0.0127 (15)	−0.0140 (14)
O2	0.0237 (16)	0.0130 (14)	0.0312 (17)	−0.0005 (12)	0.0022 (13)	0.0023 (12)
O3	0.0301 (17)	0.0167 (15)	0.0186 (15)	−0.0072 (13)	−0.0020 (12)	0.0053 (11)
N5	0.0136 (16)	0.0152 (16)	0.0143 (15)	−0.0048 (13)	−0.0033 (12)	0.0003 (12)
O4	0.044 (2)	0.0313 (18)	0.0126 (14)	−0.0133 (16)	−0.0072 (13)	0.0035 (12)
O5	0.0317 (17)	0.0230 (16)	0.0228 (16)	−0.0091 (14)	−0.0097 (13)	−0.0067 (12)
O6	0.0288 (16)	0.0150 (14)	0.0193 (14)	−0.0079 (13)	−0.0094 (12)	0.0014 (11)
N6	0.0125 (15)	0.0132 (16)	0.0156 (16)	0.0001 (13)	−0.0039 (12)	−0.0016 (12)

### Geometric parameters (Å, º)

**Table:**

Cu01—O1W	2.184 (3)	C9—C10	1.410 (6)
Cu01—N1	2.010 (3)	C11—C12	1.448 (6)
Cu01—N2	2.042 (3)	C13—C14	1.409 (6)
Cu01—N3	2.034 (3)	C14—C15	1.375 (6)
Cu01—N4	2.006 (3)	C15—C16	1.416 (6)
O1W—H1W	0.7900	C16—C17	1.439 (5)
O1W—H2W	0.8700	C16—C23	1.404 (6)
O1—N5	1.242 (4)	C17—C18	1.364 (6)
O2—N5	1.279 (5)	C18—C19	1.439 (6)
O3—N5	1.249 (4)	C19—C24	1.406 (5)
O4—N6	1.241 (4)	C19—C20	1.413 (6)
O5—N6	1.245 (5)	C20—C21	1.382 (6)
O6—N6	1.272 (5)	C21—C22	1.408 (5)
N1—C1	1.340 (5)	C23—C24	1.441 (6)
N1—C11	1.363 (5)	C1—H1	0.9300
N2—C12	1.358 (5)	C2—H2	0.9300
N2—C10	1.328 (5)	C3—H3	0.9300
N3—C13	1.337 (5)	C5—H5	0.9300
N3—C23	1.366 (5)	C6—H6	0.9300
N4—C22	1.337 (5)	C8—H8	0.9300
N4—C24	1.367 (5)	C9—H9	0.9300
C1—C2	1.405 (6)	C10—H10	0.9300
C2—C3	1.373 (6)	C13—H13	0.9300
C3—C4	1.408 (6)	C14—H14	0.9300
C4—C11	1.408 (5)	C15—H15	0.9300
C4—C5	1.443 (6)	C17—H17	0.9300
C5—C6	1.359 (6)	C18—H18	0.9300
C6—C7	1.432 (6)	C20—H20	0.9300
C7—C8	1.414 (6)	C21—H21	0.9300
C7—C12	1.409 (6)	C22—H22	0.9300
C8—C9	1.373 (6)		
			
O1W—Cu01—N1	85.46 (14)	C13—C14—C15	120.1 (4)
O1W—Cu01—N2	101.65 (13)	C14—C15—C16	119.0 (4)
O1W—Cu01—N3	114.86 (13)	C15—C16—C23	117.3 (3)
O1W—Cu01—N4	86.34 (14)	C15—C16—C17	123.7 (4)
N1—Cu01—N2	82.61 (13)	C17—C16—C23	119.0 (4)
N1—Cu01—N3	100.51 (13)	C16—C17—C18	121.2 (4)
N1—Cu01—N4	171.78 (15)	C17—C18—C19	120.8 (3)
N2—Cu01—N3	143.48 (15)	C18—C19—C20	123.8 (3)
N2—Cu01—N4	99.69 (13)	C18—C19—C24	118.9 (4)
N3—Cu01—N4	82.40 (13)	C20—C19—C24	117.3 (4)
Cu01—O1W—H2W	118.00	C19—C20—C21	119.2 (3)
H1W—O1W—H2W	105.00	C20—C21—C22	119.8 (4)
Cu01—O1W—H1W	129.00	N4—C22—C21	122.2 (4)
C1—N1—C11	117.5 (3)	C16—C23—C24	119.8 (3)
Cu01—N1—C1	129.8 (3)	N3—C23—C16	123.5 (3)
Cu01—N1—C11	112.0 (2)	N3—C23—C24	116.7 (3)
Cu01—N2—C10	130.8 (3)	C19—C24—C23	120.2 (4)
Cu01—N2—C12	111.1 (3)	N4—C24—C19	123.4 (4)
C10—N2—C12	118.1 (3)	N4—C24—C23	116.3 (3)
C13—N3—C23	117.9 (3)	N1—C1—H1	119.00
Cu01—N3—C13	130.6 (3)	C2—C1—H1	119.00
Cu01—N3—C23	111.5 (2)	C1—C2—H2	120.00
Cu01—N4—C24	112.6 (2)	C3—C2—H2	120.00
Cu01—N4—C22	128.8 (3)	C4—C3—H3	120.00
C22—N4—C24	118.1 (3)	C2—C3—H3	120.00
O2—N5—O3	119.6 (3)	C4—C5—H5	119.00
O1—N5—O2	119.2 (3)	C6—C5—H5	120.00
O1—N5—O3	121.1 (3)	C7—C6—H6	119.00
O4—N6—O6	119.8 (3)	C5—C6—H6	119.00
O4—N6—O5	121.3 (4)	C7—C8—H8	121.00
O5—N6—O6	118.9 (3)	C9—C8—H8	121.00
N1—C1—C2	122.9 (4)	C8—C9—H9	120.00
C1—C2—C3	119.3 (4)	C10—C9—H9	120.00
C2—C3—C4	119.6 (3)	C9—C10—H10	119.00
C3—C4—C11	117.4 (4)	N2—C10—H10	119.00
C5—C4—C11	118.9 (4)	N3—C13—H13	119.00
C3—C4—C5	123.7 (3)	C14—C13—H13	119.00
C4—C5—C6	121.0 (3)	C13—C14—H14	120.00
C5—C6—C7	121.1 (4)	C15—C14—H14	120.00
C6—C7—C8	123.4 (4)	C16—C15—H15	121.00
C6—C7—C12	119.7 (4)	C14—C15—H15	121.00
C8—C7—C12	116.8 (3)	C16—C17—H17	119.00
C7—C8—C9	118.9 (4)	C18—C17—H17	119.00
C8—C9—C10	120.3 (4)	C17—C18—H18	120.00
N2—C10—C9	122.0 (4)	C19—C18—H18	120.00
N1—C11—C4	123.3 (4)	C21—C20—H20	120.00
C4—C11—C12	120.1 (4)	C19—C20—H20	120.00
N1—C11—C12	116.6 (3)	C20—C21—H21	120.00
N2—C12—C7	123.9 (3)	C22—C21—H21	120.00
N2—C12—C11	117.0 (3)	C21—C22—H22	119.00
C7—C12—C11	119.1 (3)	N4—C22—H22	119.00
N3—C13—C14	122.1 (3)		
			
O1W—Cu01—N1—C1	−75.3 (4)	C22—N4—C24—C23	178.4 (4)
O1W—Cu01—N1—C11	95.0 (3)	N1—C1—C2—C3	−0.4 (7)
N2—Cu01—N1—C1	−177.7 (4)	C1—C2—C3—C4	0.9 (7)
N2—Cu01—N1—C11	−7.4 (3)	C2—C3—C4—C5	178.8 (4)
N3—Cu01—N1—C1	39.2 (4)	C2—C3—C4—C11	−1.3 (6)
N3—Cu01—N1—C11	−150.6 (3)	C3—C4—C5—C6	−179.5 (4)
O1W—Cu01—N2—C10	99.2 (4)	C11—C4—C5—C6	0.5 (6)
O1W—Cu01—N2—C12	−77.6 (3)	C3—C4—C11—N1	1.1 (6)
N1—Cu01—N2—C10	−177.0 (4)	C3—C4—C11—C12	−178.5 (4)
N1—Cu01—N2—C12	6.2 (3)	C5—C4—C11—N1	−178.9 (4)
N3—Cu01—N2—C10	−79.3 (4)	C5—C4—C11—C12	1.5 (6)
N3—Cu01—N2—C12	103.9 (3)	C4—C5—C6—C7	−1.7 (7)
N4—Cu01—N2—C10	11.0 (4)	C5—C6—C7—C8	−178.1 (4)
N4—Cu01—N2—C12	−165.8 (3)	C5—C6—C7—C12	1.0 (7)
O1W—Cu01—N3—C13	101.9 (4)	C6—C7—C8—C9	179.6 (4)
O1W—Cu01—N3—C23	−76.7 (3)	C12—C7—C8—C9	0.5 (6)
N1—Cu01—N3—C13	12.1 (4)	C6—C7—C12—N2	−178.5 (4)
N1—Cu01—N3—C23	−166.5 (3)	C6—C7—C12—C11	1.0 (6)
N2—Cu01—N3—C13	−79.7 (4)	C8—C7—C12—N2	0.6 (6)
N2—Cu01—N3—C23	101.8 (3)	C8—C7—C12—C11	−179.9 (4)
N4—Cu01—N3—C13	−175.7 (4)	C7—C8—C9—C10	−0.5 (7)
N4—Cu01—N3—C23	5.7 (3)	C8—C9—C10—N2	−0.6 (7)
O1W—Cu01—N4—C22	−62.0 (4)	N1—C11—C12—N2	−2.3 (5)
O1W—Cu01—N4—C24	109.3 (3)	N1—C11—C12—C7	178.1 (4)
N2—Cu01—N4—C22	39.2 (4)	C4—C11—C12—N2	177.3 (4)
N2—Cu01—N4—C24	−149.6 (3)	C4—C11—C12—C7	−2.2 (6)
N3—Cu01—N4—C22	−177.7 (4)	N3—C13—C14—C15	−0.8 (7)
N3—Cu01—N4—C24	−6.4 (3)	C13—C14—C15—C16	−1.5 (7)
Cu01—N1—C1—C2	170.0 (3)	C14—C15—C16—C17	−177.4 (4)
C11—N1—C1—C2	0.2 (6)	C14—C15—C16—C23	1.8 (6)
Cu01—N1—C11—C4	−172.1 (3)	C15—C16—C17—C18	−178.2 (4)
Cu01—N1—C11—C12	7.5 (4)	C23—C16—C17—C18	2.6 (6)
C1—N1—C11—C4	−0.6 (6)	C15—C16—C23—N3	0.0 (6)
C1—N1—C11—C12	179.0 (4)	C15—C16—C23—C24	−179.3 (4)
Cu01—N2—C10—C9	−174.9 (3)	C17—C16—C23—N3	179.3 (4)
C12—N2—C10—C9	1.7 (6)	C17—C16—C23—C24	−0.1 (6)
Cu01—N2—C12—C7	175.5 (3)	C16—C17—C18—C19	−2.4 (7)
Cu01—N2—C12—C11	−4.0 (4)	C17—C18—C19—C20	−179.3 (4)
C10—N2—C12—C7	−1.7 (6)	C17—C18—C19—C24	−0.4 (6)
C10—N2—C12—C11	178.7 (4)	C18—C19—C20—C21	177.9 (4)
Cu01—N3—C13—C14	−175.9 (3)	C24—C19—C20—C21	−1.0 (6)
C23—N3—C13—C14	2.6 (6)	C18—C19—C24—N4	−177.9 (4)
Cu01—N3—C23—C16	176.5 (3)	C18—C19—C24—C23	3.0 (6)
Cu01—N3—C23—C24	−4.1 (4)	C20—C19—C24—N4	1.1 (6)
C13—N3—C23—C16	−2.2 (6)	C20—C19—C24—C23	−178.1 (4)
C13—N3—C23—C24	177.1 (4)	C19—C20—C21—C22	0.7 (6)
Cu01—N4—C22—C21	171.3 (3)	C20—C21—C22—N4	−0.4 (7)
C24—N4—C22—C21	0.4 (6)	N3—C23—C24—N4	−1.3 (5)
Cu01—N4—C24—C19	−173.1 (3)	N3—C23—C24—C19	177.9 (4)
Cu01—N4—C24—C23	6.1 (4)	C16—C23—C24—N4	178.1 (4)
C22—N4—C24—C19	−0.7 (6)	C16—C23—C24—C19	−2.7 (6)

### Hydrogen-bond geometry (Å, º)

**Table:**

*D*—H···*A*	*D*—H	H···*A*	*D*···*A*	*D*—H···*A*
O1*W*—H1*W*···O2^i^	0.79	1.92	2.709 (5)	176
O1*W*—H2*W*···O6	0.87	1.89	2.718 (5)	159
C2—H2···O1^ii^	0.93	2.57	3.313 (5)	137
C5—H5···O5^iii^	0.93	2.40	3.271 (5)	156
C6—H6···O6^iv^	0.93	2.56	3.417 (6)	154
C8—H8···O3^v^	0.93	2.50	3.194 (5)	131
C10—H10···O6^vi^	0.93	2.60	3.131 (6)	117
C14—H14···O4^vii^	0.93	2.47	3.102 (5)	125
C15—H15···O4^vii^	0.93	2.59	3.157 (5)	120
C17—H17···O2^viii^	0.93	2.49	3.363 (5)	157
C18—H18···O1^ix^	0.93	2.50	3.406 (5)	166
C20—H20···O3^ix^	0.93	2.49	3.357 (5)	155

Symmetry codes: (i) *x*+1, *y*, *z*; (ii) −*x*+2, −*y*, −*z*; (iii) *x*, *y*−1, *z*; (iv) −*x*+2, −*y*, −*z*+1; (v) −*x*+1, −*y*, −*z*+1; (vi) *x*−1, *y*, *z*; (vii) −*x*+2, −*y*+1, −*z*; (viii) −*x*+1, −*y*+1, −*z*; (ix) *x*, *y*+1, *z*.

## References

[bb1] Bruker (2006). *APEX2* and *SAINT* Bruker AXS Inc., Madison, Wisconsin, USA.

[bb2] Catalan, K. J., Jackson, S., Zubkowski, J. D., Perry, D. L., Valente, E. J., Feliu, L. A. & Polanco, A. (1995). *Polyhedron*, **14**, 2165–2171.

[bb3] Dowty, E. (1995). *ATOMS* Shape Software, Kingsport, Tennessee, USA.

[bb4] Farrugia, L. J. (2012). *J. Appl. Cryst.* **45**, 849–854.

[bb5] Nakai, H. & Deguchi, Y. (1975). *Bull. Chem. Soc. Jpn*, **48**, 2557–2560.

[bb6] Sheldrick, G. M. (2008). *Acta Cryst.* A**64**, 112–122. 10.1107/S0108767307043930 18156677

[bb7] Szpakolski, K. B., Latham, K., Rix, C. J., White, J. M., Moubaraki, B. & Murray, K. S. (2010). *Chem. Eur. J.* **16**, 1691–1696. 10.1002/chem.200901720 20013774

[bb8] Zhou, Y. H. (2011). *Huaibei Shifan Daxue Xuebao (Ziran Kexueban)*, **32**, 33.

